# Investigation of the Microstructural Evolution during Hot Stamping of a Carburized Complex Phase Steel by Laser-Ultrasonics

**DOI:** 10.3390/ma14081836

**Published:** 2021-04-07

**Authors:** Alexander Horn, Marion Merklein

**Affiliations:** Institute of Manufacturing Technology, Friedrich-Alexander-Universität Erlangen-Nürnberg, 91058 Erlangen, Germany; marion.merklein@fau.de

**Keywords:** hot stamping, phase transformation, quenching, grain growth, carburization

## Abstract

Prior carburization of semi-finished steel sheets is a new process variant in hot stamping to manufacture parts with tailored properties. Compared to conventional hot stamping processes, a complex phase typed steel alloy is used instead of 22MnB5. Yet recent investigations focused on final mechanical properties rather than microstructural mechanisms cause an increase in strength. Thus, the influence of additional carburization on the microstructural evolution during hot stamping of a complex phase steel CP-W^®^800 is investigated within this work. The phase transformation behavior, as well as the grain growth during austenitization, is evaluated by in-situ measurements employing a laser-ultrasound sensor. The results are correlated with additional hardness measurements in as-quenched condition and supplementary micrographs. The experiments reveal that the carburization process significantly improves the hardenability of the CP-W^®^800. However, even at quenching rates of 70 K/s no fully martensitic microstructure was achievable. Still, the resulting hardness of the carburized samples might exceed the fully martensitic hardness of 22MnB5 derived from literature. Furthermore, the carburization process has no adverse effect on the fine grain stability of the complex phase steel. This makes it more robust in terms of grain size than the conventional hot stamping steel 22MnB5.

## 1. Introduction

Today’s automotive industry faces various challenges. One of the biggest drivers of innovations is the desire to lower pollutant emissions by either reducing fuel consumption or by establishing new propulsion concepts. Within the last decade, especially the lightweight design of car body parts was a common approach to reach this goal. Besides conventional lightweight materials such as aluminum or fiber-reinforced plastics, steel is still utilized, in particular when considering costs [1] and life cycle assessment (LCA) [2]. In this context, the application of ultra-high strength steels is of high interest primarily concerning safety-relevant components, such as b-pillars or front bumpers [3]. Against this backdrop, hot stamping of boron-manganese steels has developed to a state-of-the-art process within recent years. This process consists out of a full austenitization of the sheets above AC_3_ temperature subsequently followed by immediate combined forming and quenching. By exceeding the material-specific critical cooling rate, a fully martensitic microstructure is achieved, which results in tensile strength above 1500 MPa [4]. There are several process variants to manufacture parts with tailored properties by hot stamping [5]. Most of them aim to improve the ductility of the final components, which can be beneficial regarding crash behavior since energy absorbance can be improved [6]. Examples for these state-of-the-art process adaptions are specific heating strategies with only localized austenitization [7] or partial quenching to reduce the cooling rate in particular areas [8]. However, with several limitations regarding the flexibility of these processes [9] as well as other disadvantages such as longer process chains [5] or thermal distortions [10], the development of an alternative approach is reasonable. The concept of tailored carburization is a new process variant, which aims to adjust the mechanical properties by locally increasing the carbon content [11]. Contrary to the other process variations, local strengthening is desired rather than improved ductility. For instance, this can be advantageous in terms of battery electric vehicles, where the battery housing is often referred to as a non-deformation zone and highest structural integrity is mandatory to prevent even small damage [12].

In the process of tailored carburization, the sheets are locally coated with carbonic material. During several hours of heat treatment, the carbon atoms diffuse in the base material. Depending on the heat treatment parameters, a specific hardness gradient is developed [13]. After carburization, a semi-finished part with a distinct carbon distribution is present, which can be conventionally hot stamped afterward. Recent investigations focused on a complex phase steel CP-W^®^800 as a base material since it meets the two requirements for this process. Firstly, it is press hardenable and its carbon content in as-delivered condition allows a further increase in strength by carburization.

Compared to the conventional hot stamping steel 22MnB5 or state-of-the-art process variants, hot stamping of a locally carburized complex phase steel exhibits higher flexibility. The mechanical properties can be adjusted in a wide range, which is not bound to any tool modifications or adjustments regarding the oven technology. The same applies to the geometrical design of the zones with different tailored properties. However, the required time for the additional heat treatment for carburization might be obstructive in terms of a large-scale industrial application. As concluded in a previous study [13], the process of combined carburization and hot stamping might be suitable especially for small batch size productions and prototyping, where high flexibility is crucial. Since these types of manufacturing are often associated with a lower degree of automation, small fluctuations of individual process steps cannot be precluded. This is especially related to the heat treatment procedure. Therefore, a robust material behavior during austenitization is advantageous. To enable a suitable process design, as well as in terms of process control, it is necessary to investigate the microstructural evolution during the hot stamping process.

Regarding the complex phase steel, several studies on the phase transformation were already undertaken. Hairer et al. [14] investigated the influence of different quenching rates between 0.6 K/s and 120 K/s. Their experiments revealed, that a mixed microstructure is present at each of their analyzed cooling rate. They concluded that faster quenching increases the martensitic phase fraction in favor of ferrite. As stated by the authors, the martensite gets auto-tempered at high cooling rates, which makes it hardly distinguishable from bainite. Comparable results can be found in the study of Kang et al. [15]. While the overall hardness of as-quenched samples is lower compared to Hairer et al. [14], the determined phase transformation behavior is similar. Furthermore, Kang et al. [15] also notice the formation of auto-tempered martensite.

As summarized by Nanda et al. [16], there are several more studies on the phase transformation behavior of complex phase-type steels. However, most of their chemical composition is different from the CP-W^®^800 investigated within this publication. Therefore, a more detailed discussion on these references is not provided.

While different studies already indicate the phase transformation behavior of the conventional CP-W^®^800 during quenching, the influence of the various carbon content after carburization is unknown. Previous investigations on the process combination of carburization and hot stamping mainly concentrated on the final mechanical properties. The mechanisms for the increase in strength are not yet analyzed in detail. In this context, especially the effect of additional carbon content on the hardenability during quenching and the impact on the hardness of martensite is of interest. Further focus lies on the evaluation of the grain growth behavior. Although complex phase steels have good fine-grain stability due to microalloying [16], the carburization process might influence grain coarsening since the carbon content is linked to the solubility of microalloying precipitates [17].

Therefore, in this study, the phase transformation during hot stamping of a complex phase steel in carburized, as well as in as-delivered condition, is investigated. The results are correlated with hardness measurements and additional micrographs. The second part of this study focuses on the analysis of the grain growth behavior since grain size can significantly influence the mechanical properties as known from the Hall–Petch relationship. The measurement of the grain size and the determination of phase transformation are conducted by employing a laser-ultrasound sensor, which allows for the in-situ evaluation of microstructural changes.

## 2. Materials and Methods

### 2.1. Methodology

This investigation focuses on the microstructural changes during the hot stamping process of a carburized complex phase steel. This refers to the grain growth behavior during austenitization as well as to the phase transformation during quenching. The influence of the additional carburization process is assessed by analyzing carburized and non-carburized samples of the respective steel alloy. To enable an overall process evaluation regarding the microstructural evolution of the carburized complex phase steel during hot stamping, the results are compared to the material behavior of the conventional hot stamping steel 22MnB5.

**Figure 1:**
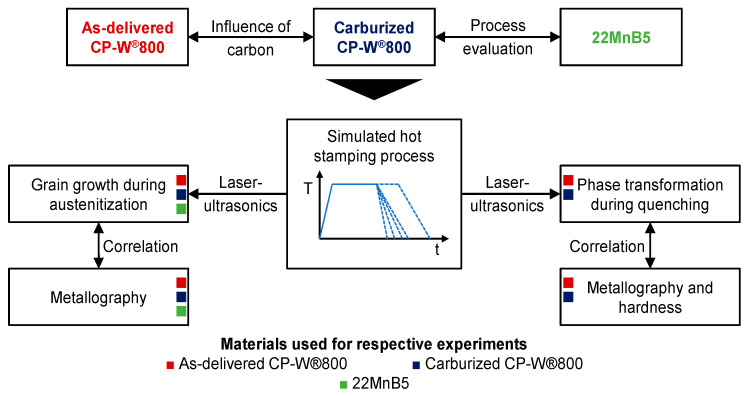
Methodology used to investigate the microstructural evolution during hot stamping of a carburized complex phase steel.

The chosen methodology within this study is shown in Figure 1. The hot stamping process is replicated with a thermophysical simulator. During the heat treatment, the grain growth behavior, as well as the phase transformation, are analyzed by employing a laser-ultrasound sensor. In terms of the phase transformation, the influence of various quenching rates will be taken into account. The results from the ultrasonic testing during simulated hot stamping is correlated with complementary investigations on secondary samples, which refers to hardness measurements and metallographic analysis.

The experiments on the phase transformation behavior are limited to the carburized and non-carburized complex phase steel since phase transformation data of 22MnB5 is broadly available in the literature. Regarding the grain growth during austenitization, the tests are done with 22MnB5 as well.

### 2.2. Materials

#### 2.2.1. Investigated Materials

The base material in this study is the complex phase steel CP-W^®^800 (Thyssenkrupp AG, Essen, Germany) with a sheet thickness of 1.6 mm. As already mentioned in the introduction, this steel grade is press hardenable and its carbon content in the as-delivered condition is significantly lower compared to the conventional hot stamping steel 22MnB5. The exact chemical compositions are shown in Table 1. Since 22MnB5 is the most common steel grade for hot stamping, the results of CP-W^®^800 are compared to those of 22MnB5. While phase transformation data is broadly available for 22MnB5, continuous grain growth behavior is not. Several studies might have analyzed prior austenite grain size depending on different austenitization parameters, however, a continuous evaluation of the grain size during holding above AC_3_ is nonexistent. Therefore, the experiments with the laser-ultrasound sensor for the in-situ characterization of the grain size is done for 22MnB5 as well for comparison.

Within this work, three different material conditions of CP-W^®^800 are investigated. The first one is the as-delivered condition. This is also referred to as the non-carburized condition and the conventional hot stamping process. To assess the influence of the additional carburization process, specimens with two different carburization treatments are tested as well. While the carburization temperature of these both conditions amounts to 900 °C, their respective carburization time is 3 h and 6 h. The parameter combinations were chosen based on previous investigations [13]. After a carburization treatment of 3 h, a distinct carbon gradient is present, while 6 h of carburization results in a more homogeneous distribution. Analyzing two material conditions with a different degree of carburization helps to improve process understanding in terms of the influence of the additional carburization on the microstructural evolution during hot stamping.

#### 2.2.2. Mechanical Properties of Carburized Complex Phase Steel

**Figure 2:**
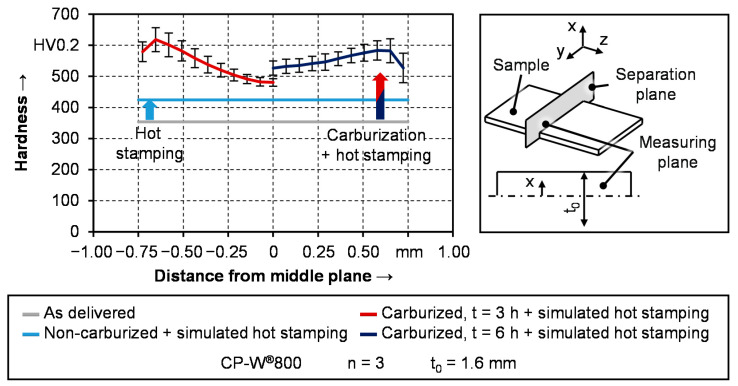
Hardness distribution in as-delivered condition as well as after hot stamping.

Regarding the complex phase steel, three different material conditions are investigated within this study. As described in the introduction section, the carburization process leads to a carbon gradient along the sheet thickness which can be visualized through the hardness distribution. Contrary to that, the samples being hot stamped from the as-delivered condition exhibit homogeneous material properties. One part of this investigation focuses on the analysis of the hardness of as-quenched samples as a function of the different material conditions and various quenching rates. For assessing the resulting hardness, it is necessary to set up a benchmark for each material condition. For this purpose, the hardness of carburized and non-carburized samples after imitated hot stamping is shown in Figure 2. Each of these samples underwent austenitization for 4 min at 900 °C. To simulate the hardening step in hot stamping, the sheets were water quenched after austenitization. Since specimens without carburization do not exhibit a hardness gradient, the values are presented as a horizontal line. In the as-quenched condition, the hardness of non-carburized specimens amounts to around 420 HV0.2. This is in good agreement with a previous study from Merklein and Svec [20], where a suitable process window for the austenitization of CP-W^®^800 was defined on basis of hot stamping experiments. In their work, the authors used a special quenching tool for their experiments. Since the hardness of water-quenched and tool-quenched samples are coincident, the approach with water cooling for simulated hot stamping is suitable.

Due to the symmetry of the carbon diffusion process, the hardness profile of the carburized samples presented in Figure 2 is only shown from the mid-section (x = 0 mm) to the edge (x = 0.8 mm). It can be seen that after 6 h at 900 °C that the hardness gradient is a significantly smaller compared to the treatment of 3 h so that the hardness distribution is more homogeneous. This can also be seen through the carburization depth, which is often referred to as the distance from the surface, where a hardness of 550 HV is achieved [21]. In the case of the 3 h at 900 °C, the carburization depth amounts to 0.4 mm and in the case of 6 h, nearly complete through hardening is reached with respect to the standard deviation. The resulting hardness ranges between 480 HV0.2 and 620 HV0.2 after 3 h of carburization and from 520 HV0.2 to 580 HV0.2 after 6 h of carburization.

### 2.3. Experimental Methods

#### 2.3.1. Simulation of the Thermal Treatment during the Hot Stamping Process

The experiments for the investigation of the microstructural evolution are done in a thermophysical simulator Gleeble 3500 GTC from Dynamic Systems Inc. (Poestenkill, NY, USA). to simulate the actual hot stamping process. The sheets are heated with a heating rate of 15 K/s to a temperature of 900 °C and held for 4 min. To replicate the in-die quenching step of conventional hot stamping processes, the samples are cooled with compressed air after austenitization. To investigate the cooling rate dependent phase transformation behavior, nominal quenching rates of 10 K/s, 30 K/s, 50 K/s, 70 K/s, and 100 K/s are applied. The temperature is controlled using point welded thermocouples type K. The nominal cooling rate is constant upon the beginning of phase transformation. Due to latent heat during phase transformation, the effective quenching rate is lower. The resulting average quenching rates in the temperature range between 800 °C and 250 °C are listed in Table 2. To reduce any oxidation of the samples, which significantly influences the signal-to-noise ratio during laser-ultrasonic testing, the experiments are done in a vacuum. To improve the significance of the experimental results, each parameter combination is tested three times as indicated in any figure by *n* = 3.

Besides this described process sequence, additional tests are done with longer holding times up to 6 min to estimate the sensitivity of the grain size to fluctuations of the dwell time. These supplementary tests are done with carburized samples and with the conventional hot stamping steel grade 22MnB5 for comparison.

#### 2.3.2. In-Situ Analysis of the Microstructure by Laser-Ultrasonics

**Figure 3:**
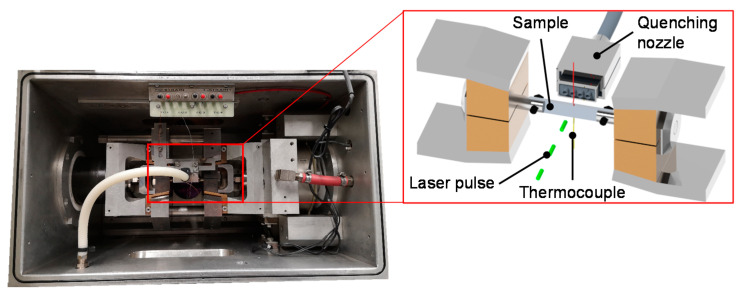
Experimental setup for the evaluation of the microstructural evolution during hot stamping employing a laser-ultrasound sensor.

The evaluation of the microstructural changes during the simulated heat treatment of the hot stamping process is done using a laser-ultrasonic technique. This measurement principle is based on the interaction between a laser-generated ultrasound impulse and the sample material [22]. For this purpose, a LUMet system from Dynamic Systems Inc. (Poestenkill, NY, USA) and Tecnar (Saint-Bruno-de-Montarville, Canada) is used, which is directly coupled to the thermomechanical simulator Gleeble 3500-GTC (Dynamic Systems Inc., Poestenkill, NY, USA). The system utilizes a frequency-doubled Nd:YAG laser pulse to vaporize a small amount of material on the sample’s surface. The vaporization depth is only in the order of around 10 nm [23]. The thermomechanical pressure on the surface due to the ablation process generates an ultrasonic pulse in the sample. This impulse travels through the sample and is reflected on the rear side. The incoming echo on the front is then detected by a laser interferometer. This happens not only for the first but also for the consecutive echoes. The measured ultrasound signal represents the average characteristics of the measuring volume, which consists out of the laser spot with a diameter of around 2 mm times the sample’s thickness. The experimental setup is shown in Figure 3.

The determined ultrasound signal has two measurands that can be used for the characterization of the material’s properties. The ultrasonic velocity is influenced by the elastic moduli [24], which is itself affected by other factors such as temperature, alloying and phase transformation [25]. In their review on the correlation between ultrasonic properties and microstructural evolution, Toozandehjani et al. [22] presented several empirical formulas for the relationship between the elastic constants and the ultrasonic velocity. During ultrasonic testing, the velocity is identified from two consecutive echoes of one ultrasound signal. From the temporal delay between those echoes and the known sample thickness, which corresponds to the covered distance, the velocity can be calculated. Since other factors such as alloying are constant during experiments or can be directly measured like temperature, the velocity can be used to detect phase transformations. The applicability of this method was already validated by several studies. In the investigation of Militzer et al. [26], the results from ultrasonic testing show good agreement with conventional dilatometry. Dubois et al. [25] validated the mathematical relationship by comparing the measured velocity with calculated values. Kruger and Damm [27] investigated different steels with various carbon content and concluded that the carbon content of the alloy has only a negligible effect on the velocity. However, in a further study, Kruger et al. [28] also noticed a deviation between dilatometry and laser ultrasonics during an isothermal phase transformation into bainite. The authors refer to this behavior as a possible carbon enrichment of austenite, which influences elastic constants [29].

The second measurand is the attenuation of the amplitude between consecutive echoes, which is caused by scattering, diffraction, absorption, and reflection. In polycrystalline materials, the dominant mechanism scatters at grain boundaries. Depending on the ratio between the acoustic wavelength and the grain size, the relationship between grain size and attenuation is either a direct or an indirect proportional [30]. The evaluation of the ultrasound signal is done with the related software CTOME^®^ (V2.31.03, 2021, CTOME Software & Consulting Inc., Vancouver, Canada). Compared to conventional methods, such as a contact dilatometer and metallographic analysis, laser-ultrasonics has several advantages. Especially in systems with resistance heating, a temperature gradient influences the measurement of phase transformation. This phenomenon is even more apparent when using a contact linear variable differential transducer (LVDT), where air-cooled quartz rods are in contact with the heated sample. On the contrary, laser-ultrasonics is based on a small measuring volume, where constant temperature can easily be achieved. Regarding the evaluation of grain size, it is possible to do continuous in-situ measurements at elevated temperatures with up to 50 Hz. Conventional metallographic analysis would require a significant amount of sample preparation work to get a fraction of measurement density. This measuring principle was already used and validated for several applications. Garcin et al. [31] investigated the influence of prior austenitic grain size and phase on the growth during reheating. In their work, Militzer et al. [26] measured inter alia the phase transformation and grain development during the simulation of a dual torch welding process. While these studies focus on steel, also other materials can be evaluated, as seen with titanium by Shinbine et al. [23] or superalloys by Garcin et al. [32].

#### 2.3.3. Supplementary Experiments

After the simulated hot stamping process in the thermophysical simulator Gleeble GTC (Dynamic Systems Inc., Poestenkill, NY, USA), secondary samples are taken from the heat-treated specimens for further investigations. This includes microhardness measurements using a Fischerscope HM2000 (Helmut Fischer GmbH, Sindelfingen, Germany) and metallographic analysis. Regarding the microscopy work, the samples are etched with 3% Nital for the qualitative identification of different phases as well as with the etchant “Grün QT” from Schmitz Metallographie GmbH (Herzogenrath, Germany) to make prior austenitic grain boundaries visible.

## 3. Results and Discussion

### 3.1. Phase Transformation during Hot Stamping

#### 3.1.1. Procedure for the Evaluation of the Testing Results

**Figure 4:**
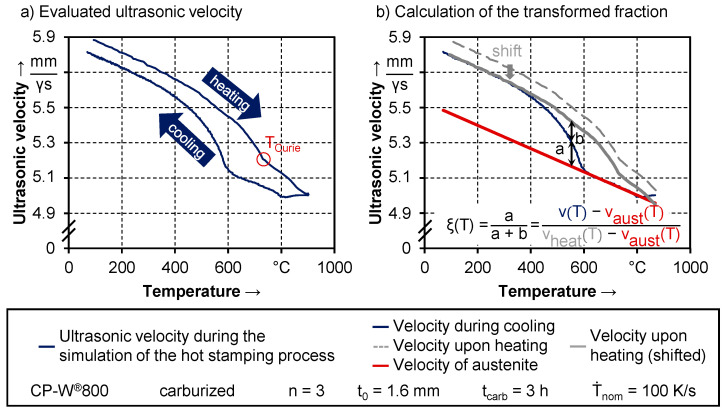
(**a**) Results from the measured ultrasonic velocity and (**b**) calculation of the transformed fraction.

The first part of the results section focuses on the phase transformation during hot stamping depending on various quenching rates and material conditions. This is done by comparing the temperature-dependent decomposition of the austenitic phase, as well as by determining the beginning and end of the phase transformation. The calculation of the transformed fraction ξ is done from the resulting data from the ultrasound evaluation computed in the CTOME^®^ software. In Figure 4a, the development of the ultrasonic velocity during the simulation of the hot stamping process in the Gleeble is depicted.

The system continuously measures the velocity from 75 °C up to 900 °C during heating and austenitization and until 100 °C during quenching. From this data, two important aspects are visible. During heating, the change in velocity is non-linear. Furthermore, there is a distinctive change in slope at a temperature of around 770 °C, which is equal to the Curie temperature of the α-phase. For higher temperatures, the velocity of α changes linearly. For the austenitic phase, the linear correlation between temperature and velocity is valid for all temperatures. Since the velocity of the γ-phase and the α-phase only exhibit minor differences above the Currie temperature of α and both vary linearly, phase transformations are barely detectable in this temperature range [27]. This has to be taken into account when trying to investigate phase transformations. Since previous investigations from Hairer et al. [14] and Kang et al. [15] on the current CP-W^®^800 suggest that the onset of phase transformation is at around 700 °C, the ultrasonic measurement method is applicable.

Another conclusion that can be derived from the change in slope of the ultrasonic velocity above T_Curie_ is that the classical lever rule with two tangents cannot be applied, since the velocity of the α-phase shows a non-linear dependence. Therefore, an approach proposed by Militzer et al. [26] is used, where a lever rule between the tangent of the linear varying austenitic phase and the non-linear varying α phase, measured during heating, is applied. As presented in Figure 4b, a tangent is fitted in the high-temperature range before the onset of phase transformation of the ultrasonic velocity curve during quenching as in conventional dilatometry. This corresponds to the ultrasonic velocity of the austenite v_aust_(T). To account for the non-linearity of the α-phase, the measured curve upon heating is shifted to match the ultrasonic velocity curve measured during quenching in the low-temperature range. The transformed fraction ξ is calculated according to the lever rule shown in Figure 4b.

#### 3.1.2. Phase Transformation of Non-Carburized CP-W^®^800 during Conventional Hot Stamping

**Figure 5:**
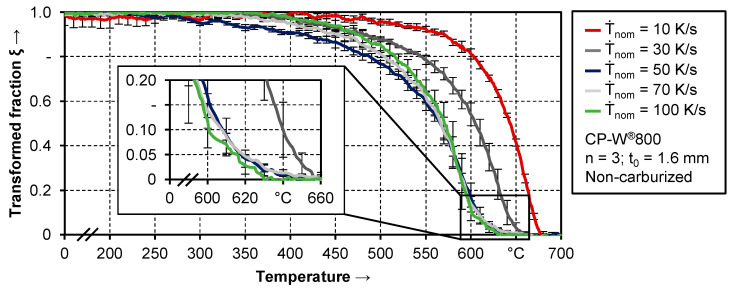
Decomposition of the austenitic phase during quenching of non-carburized samples.

Figure 5 shows the decomposition of the austenitic phase during the quenching of non-carburized samples. Nominal cooling rates between 10 K/s and 100 K/s were applied. An increase in cooling speed reduces the onset of phase transformation. For 10 K/s, the decomposition of austenite begins roughly at 675 °C. For higher quenching rates above 30 K/s, only a minor effect can be seen, which is not significant due to the standard deviation.

Another difference is visible regarding the slope of the decomposition curve. For 10 K/s, 30 K/s, and 50 K/s the curve progression is very similar, where the respective graphs exhibit a nearly constant shift in temperature between each other. Comparing 50 K/s and 70 K/s, different behavior is observable. In the high-temperature range at the beginning of the transformation, the graph for 70 K/s is shifted to lower temperatures, as already seen for quenching rates between 10 K/s and 50 K/s. At around 50% transformed fraction, the slope in the decomposition curve increases for a cooling rate of 70 K/s, so that the transformation is finished at a higher temperature. This progress is even more apparent for a quenching rate of 100 K/s, where the change in slope already begins at a transformed fraction of around 0.3. These differences regarding the transformation rate might be an indication of significant changes regarding the phase composition.

Various authors already analyzed the transformation behavior of complex phase steels, as seen in the review of Nanda et al. [16]. When comparing the results of these investigations to the current study, varying phase compositions have to be taken into account, especially since the exact alloying system is not listed completed in any case. Furthermore, differences regarding the heat treatment have an additional influence. However, quite similar material behavior can be seen. In the work of Hairer et al. [14], quenching rates from 0.6 to >80 K/s were investigated. The onset of phase transformation is slightly higher compared to the current results but it is in the same range of approx. 600 °C to 700 °C and shows the same trend. A very good agreement can be observed with the results published by Kang et al. [15]. This relates to the decrease in temperature regarding the onset of phase transformation as well as the transformation rate.

#### 3.1.3. Comparison of Start and End Temperatures of the Phase Transformation of Carburized and Non-Carburized Semi-Finished Parts during Simulated Hot Stamping

**Figure 6:**
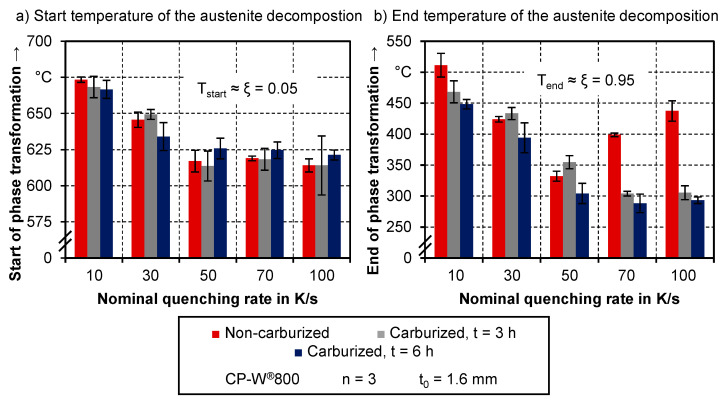
(**a**) Start and (**b**) end temperature of the austenite decomposition.

For a more quantitative analysis, the beginning and end of the phase transformation were calculated from the decomposition curves for non-carburized as well as carburized samples. Figure 6 shows the respective values, whereby the start of phase transformation equals a transformed fraction of 5% and the end of transformation equals 95%. There is no significant effect of the material condition in terms of the beginning of phase transformation. For all three tested conditions, the onset of phase transformation is shifted to lower temperatures with an increasing quenching rate. However, the difference in the start temperature amounts to less than 75 °C between cooling rates of 10 K/s and 100 K/s. The overall high start temperatures are an indication, that a ferritic phase fraction is present for all three material conditions.

Regarding the conventional hot stamping alloy 22MnB5, different studies suggest that a comparatively low quenching rate is sufficient to prevent the formation of ferrite. Aranda et al. [33] presented a CCT (continuous cooling transformation) diagram, which indicates a necessary cooling rate of 10 K/s to have a solely bainitic and martensitic phase composition. In their work, Nikravesh et al. [34] identified a cooling rate of 16 K/s to prevent the formation of ferrite during quenching of non-deformed 22MnB5. Comparable results can be found in the investigation of Horn et al. [35]. While the transformation curves upon quenching with 10 K/s exhibit a ferritic transformation, those with a cooling rate of 20 K/s do not. The significantly bigger driving force for ferrite formation in CP-W^®^800 can be explained by the differences in Si content. Silicon boosts the nucleation rate of ferrite since it enhances the carbon diffusivity in austenite by preventing the formation of carbides [36].

Regarding the end of phase transformation, which equals a transformed fraction of 0.95 in this case, there are more pronounced differences between the respective material conditions. Within the investigated cooling rates, the carburized samples exhibit a decreasing finish temperature with increasing quenching rates.

Between nominal rates of 70 K/s and 100 K/s, the change in T_end_ is not significant due to the standard deviation, which can be linked to the low differences regarding the effective cooling rate shown in Table 2. For all cooling rates, samples with 6 h of carburization exhibit the lowest finish temperature for phase transformation. Under consideration of the standard deviation, the lowest value of around 300 °C is already reached at a quenching rate of 50 K/s. In the case of semi-finished parts with three h of carburization, the descent to the minimum of T_end_ takes until a cooling rate of 70 K/s. 

Finish temperatures below 400 °C are normally associated with the formation of martensitic phase fractions. Therefore, in the case of carburized samples, martensite is expectable for quenching rates of at least 50 K/s. However, it must be considered, that the measured phase transformation behavior is an average of the measuring volume. In the case of the carburized samples, this refers to different sections with various carbon content. A more detailed analysis of this is done through the hardness measurements in Section 3.2.

Regarding the hot stamped samples from the as-delivered condition, at a cooling rate of 10 K/s, the phase transformation ends at a significantly higher temperature compared to the carburized material condition. With an increasing quenching rate, this end temperature decreases to a minimum at a cooling rate of 50 K/s and goes up again for further increase in the quenching rate. In the case of cooling rates of 30 K/s and 50 K/s, the phase transformation finishes at a temperature, which is even below the end temperature of samples with prior carburization of 3 h.

As previously stated, low finish temperatures are normally associated with the formation of martensite. However, the significant increase in T_end_ for cooling rates above 50 K/s suggests something else. Moreover, as later results from hardness measurements and metallographic analysis will show, no martensitic phase fractions are detectable in the as-quenched condition of as-delivered samples. Therefore, the drop in phase transformation finish temperature is linked to another factor than martensitic transformation. More likely than this is carbon enrichment during the ferrite transformation, which increases the carbon content of the remaining austenite. Due to the higher carbon content, the formation of subsequent phases such as bainite is shifted to lower temperatures [37]. This effect is present at low cooling speeds, where a significant amount of ferrite is likely to form. With higher cooling rates, the amount of ferrite is expected to decrease and therefore, the effect of the carbon enrichment decreases. As a result, the transformation process is shifted back to higher temperatures [38]. Kang et al. [15] as well as Hairer et al. [14] also observe carbon enrichment in their investigations on the transformation behavior on complex phase typed steel alloys.

In this section, the influence of the additional carburization on the start and end temperatures of phase transformation during hot stamping was shown. The measurement data revealed, that the carburization process does not have an impact on the start temperature, while the end temperature is significantly lowered. Compared to the non-carburized samples, the lowering of the end temperature was associated with a shift of the phase composition towards harder fractions rather than carbon enrichment. For a more detailed analysis, it is necessary to evaluate not only the start and end temperature but also the whole progress of the decomposition of austenite.

#### 3.1.4. Comparison of the Austenitic Decomposition of Carburized and Non-Carburized Semi-Finished Parts during Simulated Hot Stamping

**Figure 7:**
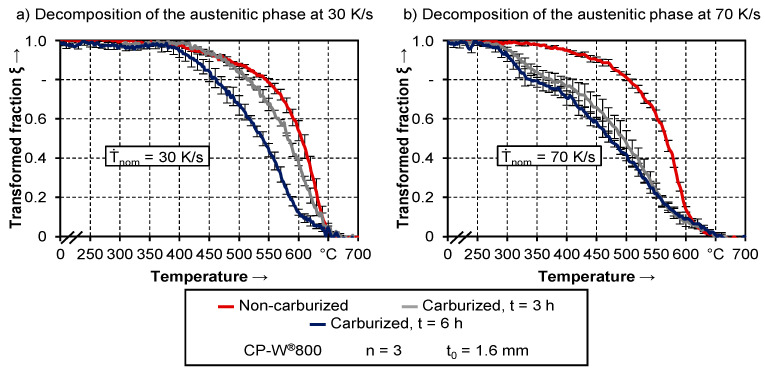
Decomposition of the austenitic phase at a quenching rate of (**a**) 30 K/s and (**b**) 70 K/s for all three different material conditions.

Besides the start and end temperatures of the phase transformation, also the curvature of the fraction transformed shows clear differences between the three material conditions. Figure 7 exemplarily shows the development of the austenite decomposition depending on two different quenching rates.

While the beginning of the phase transformation is nearly identical for all three semi-finished parts and both quenching rates, the transformation curve is shifted to lower temperatures. Due to the additional carburization process and the increased carbon content, the austenitic phase is thermally stabilized [39]. In the case of a quenching rate of 30 K/s, there were only minor differences between the material conditions regarding T_end_. After 3 h of carburization, the shift of the transformation curves is only small and no changes in the curvature are observable. Regarding samples with 6 h of carburization, the shift is more pronounced. Furthermore, the phase transformation activity is only small above 600 °C. This can be associated with the formation of less ferrite compared to the other two material conditions.

**Figure 8:**
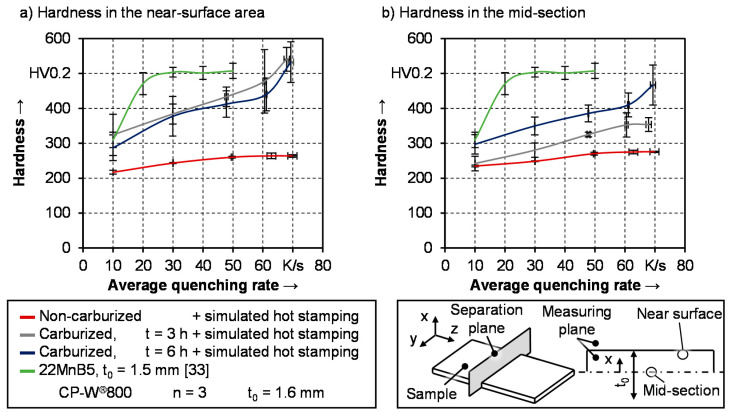
Development of Vickers hardness in the (**a**) near the edge and (**b**) in the mid-section as a function of the average quenching rate.

While the differences between the curves are mainly limited to the beginning of the phase transformation at 30 K/s, the whole curvature is affected at a quenching rate of 70 K/s. Again, the expected ferrite transformation above temperatures of 550 °C is less pronounced for the carburized samples. The subsequent formation of bainite presumably ends at a transformed fraction of 0.6. This is linked to a distinct change in the curvature at a temperature of around 380 °C. Considering the resulting hardness values in Figure 8, this can be associated with the martensite start temperature.

As might have been indicated from the results in Figure 7, the carburized samples exhibit a cyclic behavior. At a quenching rate of 10 K/s, the decomposition of the austenitic phase is similar for both carburization times. With increasing cooling rates, both curves diverge from each other first but then converge again. This can be traced back to the pronounced gradient in carbon content due to insufficient carburization time after three h. As presented in Figure 2, especially in the mid-section of the samples, the hardness difference between conventionally hot stamped specimens and those being additionally carburized for three h amounts to only 50 HV. The transformation behavior of these only slightly carburized areas is more like the behavior of the as-delivered material condition, while the outer areas with higher carbon content exhibit different behavior.

At a low quenching rate of 10 K/s, the hardenability of neither the mid-section nor the outer area is sufficient for a significant variation in the transformation behavior. With a faster cooling rate of 30 K/s, a higher degree of carburization leads to a change in the decomposition of the austenitic phase. In the case of specimens being carburized for 3 h, this applies only to the edge region with more elevated carbon content, so that there is a gradient regarding the transformation kinetics. The measurement principle of laser-ultrasonics delivers an average value of the measuring volume, which includes areas with a low and high degree of carburization. As a result, the calculated decomposition of the austenitic phase of these samples is approximately an average between the as-delivered condition and the fully carburized condition after a heat treatment of 6 h. At even higher cooling rates, the degree of carburization in the mid-section of samples being carburized for three h is then sufficient to change the transformation behavior as well. Therefore, with increasing quenching speed, the respective curves of both carburized conditions converge again.

For a suitable classification of the phase transformation behavior of the carburized and the as-delivered CP-W^®^800, the conventional hot stamping steel 22MnB5 is used as a reference. Its transformation behavior was already widely investigated by various authors, such as Naderi [40], Barcellona et al. [41], and Nikravesh et al. [42]. Compared to the data available in the literature for the 22MnB5, the experimentally determined phase transformation curves in this study indicate a significantly lower hardenability of CP-W^®^800, both in as-delivered and carburized condition. The critical quenching rate for 22MnB5 amounts to approximately 27 K/s [33] for a fully martensitic microstructure.

The respective martensite start temperature is around 400 °C [43]. While the transformation curves of the conventionally hot stamped CP-W^®^800 did not exhibit any signs of martensitic transformation, the martensite start temperature of carburized samples was in a comparable temperature range. However, within the investigated quenching rates, the onset of phase transformation for all material conditions was above 600 °C, which is associated with the existence of ferritic phase fractions. Therefore, the results of the transformation curves indicate, that a fully martensitic microstructure was not achievable for the complex phase steel within the given range of cooling rates, neither in as-delivered condition nor after additional carburization.

Through the decomposition of the austenitic phase, it was shown, that the additional carburization process leads to a shift of the phase transformation behavior towards lower temperatures and therefore probably harder phases. The results revealed that the effect is more pronounced for longer carburization times. This was justified with a higher degree of carburization. In this context, the increased stability of the austenitic phase due to the elevated carbon content has to be mentioned. By means of the two decomposition curves of the carburized samples in Figure 7, it could be seen that the additional stability of austenite lowers the phase transformation temperatures. However, a more detailed analysis of this behavior is rather challenging on basis of the present experimental data, since none of these samples exhibits a homogeneous carbon content. The measured ultrasonic velocity corresponds to an average value of the measuring volume. Therefore, different layers with various carbon content are included in this data. Moreover, possible effects of carbon enrichment on the ultrasonic velocity are neglected within this analysis. While Kruger and Damm [27] concluded that the carbon content of the respective alloy only has a negligible effect, Kruger et al. [28] noticed a possible influence of carbon enrichment during isothermal phase transformation. In future investigations, the effect of additional carbon enrichment could be taken into account, i.e., by adapting the approach suggested by Kop et al. [44].

### 3.2. Hardness of As-Quenched Samples

After quenching, secondary samples were taken from the heat-treated samples and the hardness of the hot stamped specimens was measured. To account for the variable hardness gradient along the sheet thickness of carburized samples, the hardness was evaluated in the mid-section and 200 μm below the surface. The results are depicted in Figure 8, whereby the cooling rates on the x-axis correspond to the effective values and not to the nominal rates. For a suitable comparison of CP-W^®^800 to the conventional hot stamping steel 22MnB5, its hardness values in as-quenched conditions derived from literature are included as well. In this context, it is noteworthy, that the hardness values in the mid-section and near the surface are the same for 22MnB5 since no hardness gradient is existent.

There are distinct differences regarding the condition of the semi-finished parts and in terms of the measurement area of CP-W^®^800. Within the investigated cooling rates, there is only a small effect on the resulting hardness of as-quenched samples from the as-delivered condition. While there is an increase of 20 HV0.2 between cooling rates of 30 K/s and 50 K/s, for faster quenching rates, the hardness remains around 270 HV0.2 to 275 HV0.2. This is in good agreement with previous findings from Figure 5, where the decomposition of the austenitic phase showed only minor differences for nominal quenching rates between 50 K/s and 100 K/s. Moreover, the drop in transformation finish temperature at 50 K/s was explained with carbon enrichment rather than the formation of martensite. The hardness values do support this since the formation of martensitic phase fractions would significantly increase the hardness. Considering the average quenching rates between 800 °C and 250 °C, the results are in good accordance with the measured values from Kang et al. [15]. However, after being heat-treated with the described parameters, the samples have a lower hardness compared to the water quenched samples, as well as in the as-delivered condition shown in Figure 2.

Contrary to these samples, previously carburized semi-finished parts exhibit a strong influence on the quenching rate. Within this context, it must be distinguished between both measurement areas, especially for samples being heat-treated for 3 h. At the measuring spot 200 μm below the surface, the hardness shows higher values compared to the mid-section. This can easily be attributed to the increased carbon content in this area since carbon diffusion is directed from the surface to the mid-section. The hardness increases more rapidly above averaged quenching rates of 60 K/s, which corresponds to the nominal quenching rates of 70 K/s and 100 K/s. This is in good agreement with the results from the ultrasonic phase transformation measurements, where the transformation curves indicated the formation of martensitic phase fractions. Increasing the holding time for carburization from 3 h to 6 h, a homogeneous carbon distribution is present in the material. As a result, the hardness and influence of the quenching rate are identical in the mid-section and near the surface. Contrary to that, after 3 h of carburization, the mid-section has a significantly lower carbon content, which ranges between the as-delivered condition and the six-hour carburization condition. Still, the increase in carbon by additional carburization is sufficient to improve hardenability. In the case of the samples in the as-delivered condition, the transformation curves indicated a mainly ferritic phase composition with small amounts of bainite, which is in good agreement with the resulting hardness. Due to the prior carburization of three h at 900 °C, the phase transformation is shifted towards harder phases. As a result, the increase in hardness with a rising quenching rate is significantly more pronounced in the case of the carburized samples. However, the carbon content in the mid-section for these samples is insufficient for the formation of martensite. While samples with 6 h of carburization exhibit a pronounced increase in hardness in the mid-section for an averaged quenching rate above 60 K/s, the ones with 3 h of carburization run into a plateau.

The resulting hardness of the samples in as-quenched condition show, that the hardenability of the complex phase steel can be significantly improved by prior carburization. Still, the phase transformation curves shown in Section 3.1.4 indicated that an average quenching rate of up to 70 K/s is insufficient for a complete martensitic microstructure. Compared to the conventional hot stamping steel 22MnB5, the overall hardenability is lower. At a quenching rate of 10 K/s, the hardness of 22MnB5 is in the same range as the hardness below the surface of the carburized CP-W^®^800. Increasing the quenching rate to 20 K/s and further to 30 K/s significantly improves the hardness of 22MnB5, while only a slow rise can be seen in the case of the carburized complex phase steel. This is directly linked to the growth of the martensitic phase fraction of 22MnB5 after hot stamping [35]. Further acceleration of the quenching process does not entail an additional enhancement of the hardness, as shown in Figure 8. In contrast to this, the carburized complex phase steel shows a continuous increase in hardness with rising cooling speed. A sufficient quenching rate assumed that after 6 h of carburization at 900 °C the hardness of the complex phase steel exhibits values of around 530 HV0.2 near the surface and 460 HV0.2 in the mid-section. This hardness is achieved, although a mixed microstructure is present. This can be explained by the fact that an increase in carbon not only improves the hardenability in terms of the transformation kinetics but also increases the hardness of the martensite as well [45]. As a result, the as-quenched hardness of the carburized complex phase steel is comparable to the hardness of the fully martensitic 22MnB5 even though other phases like ferrite and bainite are coexistent in CP-W^®^800.

Especially in the case of carburized samples with 6 h of heat treatment, the development of the hardness suggests, that even higher values are achievable with a further increase of the quenching rate. The hardness values of water quenched samples shown in Figure 2, which can be seen as the benchmark in terms of the achievable hardness, confirm this assumption. At a measuring spot of 200 μm below the surface, carburized samples exhibit a hardness of around 615 HV and approximately 580 HV. This is slightly higher compared to the results from Figure 8 with controlled cooling rates between 10 K/s and 70 K/s. Regarding the hardness in the mid-section, the differences are bigger. After water quenching, the resulting hardness of carburized samples is 130 HV0.2 and 60 HV0.2 higher compared to the values in Figure 8. In the case of the as-delivered material condition, the increase in hardness after water quenching is even more pronounced. While the maximum hardness amounts to around 260 HV0.2 in Figure 8, values of 420 HV0.2 are presented in Figure 2. It can be derived from the results in Figure 2 and Figure 8 that a further acceleration of the cooling process will increase the hardness beyond the values identified within the conducted experiments. However, due to latent heat, it was not possible to investigate higher average quenching rates under controlled test conditions within this study.

### 3.3. Metallographic Analysis

**Figure 9:**
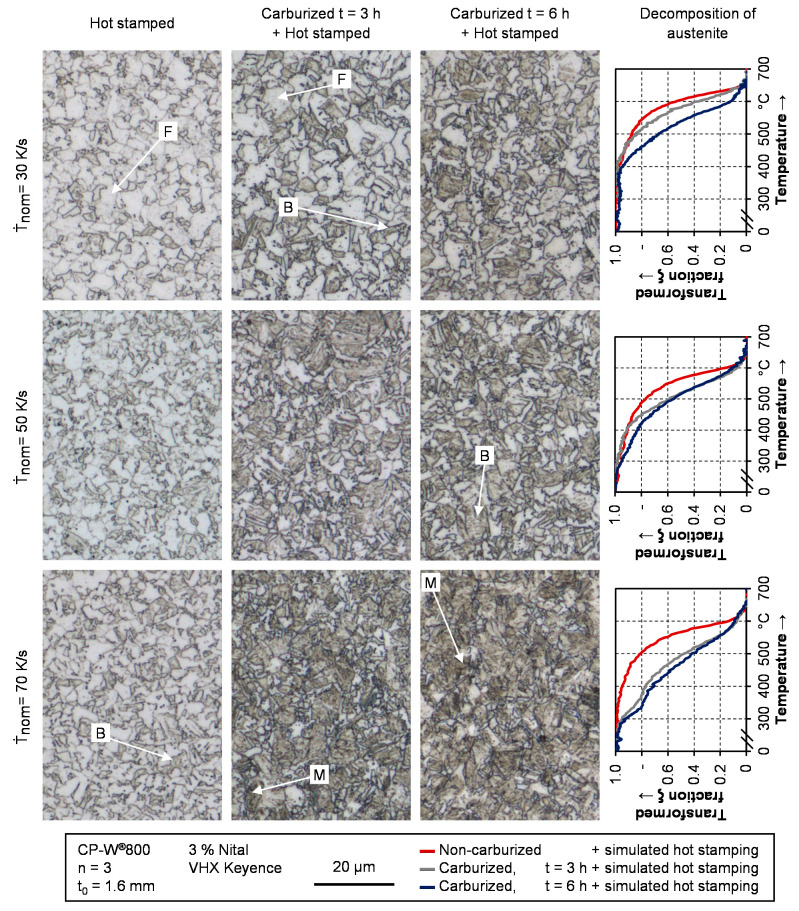
Etched micrographs of carburized and non-carburized samples after quenching with different cooling rates.

After analyzing the hardness of carburized and non-carburized samples after quenching, a qualitative evaluation of the microstructure is conducted. Figure 9 shows the micrographs after etching with Nital. Three different nominal quenching rates of 30 K/s, 50 K/s, and 70 K/s are depicted. To enable a suitable classification of the results, the decomposition of the austenitic phase is depicted as well for each cooling rate. To improve readability, no standard deviation is shown. However, the repeatability of the experimental results is in the same range as apparent from Figure 7.

As expected, there are distinctive differences between the three material conditions and the various quenching rates. Regarding the samples being hot stamped from the as-delivered condition, only small changes in the microstructure are visible. As derived from the phase transformation curves and the hardness measurements, the microstructure is mainly composed of ferrite (F). Increasing the quenching rate, areas with bainite (B) also occur. Martensitic phase fractions are not detectable, which is in good agreement with previous findings. Before hot stamping, the as-delivered samples exhibited a mixture of bainite, tempered martensite and ferrite [46]. The lack of martensite also explains the fact that the hardness in the as-quenched condition of these samples is below the hardness in as-delivered condition before hot stamping, depicted in Figure 2.

For samples being carburized for 3 h before hot stamping, the microstructure also exhibits a lot of ferrite for a nominal quenching rate of 30 K/s. Since these micrographs were taken in the mid-section of the samples, this was expectable from the hardness values. In the case of the sheets with 6 h of carburization, the amount of the bainitic phase fraction is significantly higher. These findings are in good agreement with the assumptions derived from Figure 7. For a cooling rate of 50 K/s, the amount of ferrite decreases in favor of bainite (B) and possibly tempered martensite, which is hardly distinguishable [47]. As expected from the transformation curves in Figure 7, the micrographs of the carburized sheets in Figure 9 show martensitic structures for a nominal quenching rate of 70 K/s.

As already derived from the phase transformation curves and the hardness values, the micrographs also confirm the decreased hardenability of the complex phase steel in comparison with 22MnB5. For all presented micrographs, a mixed microstructure with soft phases like ferrite is present. In contrast, the 22MnB5 exhibits a fully martensitic microstructure for cooling rates of at least 27 K/s [33].

### 3.4. Assessment of Grain Growth

Besides the phase composition, the grain size must be considered in terms of a microstructural analysis. This is even more apparent regarding the carburization process lasting for hours and its influence on the austenitization process. Within this work, especially the grain growth during the hot stamping process is evaluated. For this purpose, the laser-ultrasound sensor is utilized as well. The grain growth is assessed through ultrasonic attenuation. The attenuation of an ultrasound signal traveling through the material is caused by either grain scattering, diffraction, or internal friction [48]. Depending on the ratio between grain size and ultrasonic wavelength, the scattering of the ultrasonic signal can be associated with one of three different regimes, which influences the relationship between the attenuation coefficient and the grain size [49]. A more detailed description of the procedure for evaluating the grain scattering associated attenuation from raw data under consideration of other sources for attenuation can be found in [32]. The calculated grain size from ultrasonic measurement data corresponds to the average value of the cylindrical measuring volume with the laser spot as base area and the sheet thickness as height.

**Figure 10:**
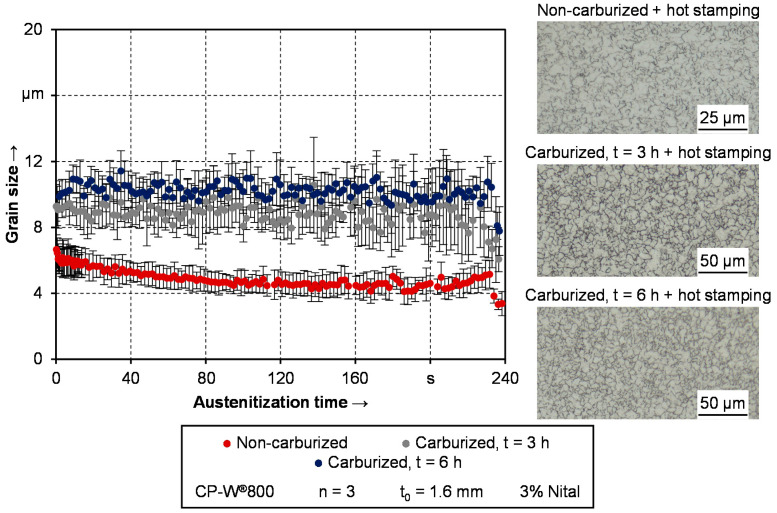
Development of the grain size during austenitization from as-delivered and carburized condition.

Figure 10 shows the grain size development of the three different material conditions during the austenitization phase of the preceding quenching test. For a qualitative validation of the grain size, micrographs of etched samples are included in the depiction. A time of 0 s corresponds to the beginning of the holding time, after heating to the austenitization temperature of 900 °C.

There is no grain growth neither in the as-delivered nor in the carburized condition. This can be attributed to the microalloying elements like vanadium, titanium, or niobium. These alloys retard grain growth due to the solute drag and Zener-drag effect [50]. Especially the formation of carbides such as NbC exerts a pinning pressure on the grain boundaries, as concluded in various studies such as [51] or [52]. The measured grain size during austenitization is in good agreement with the results from Militzer et al. [53]. In their study, the grain size of the investigated complex phase typed steel was on a constant level of around 5 μm during 15 min of holding at 900 °C.

While the overall grain size of the carburized samples is higher, neither a distinct influence of the carburization time nor an influence of the austenitization time is observable. Even though grain growth appears during the several hours of carburization, the grain size is in the range of 10 ± 2 μm throughout the whole austenitization process. This can be linked to a grain refinement during the α→μ transformation upon heating [54]. The austenite grains primarily form at previous austenitic grain boundaries [55] and packet and block boundaries within martensite [56]. Regarding the micrographs of the etched samples included in Figure 10, the results determined from the laser-ultrasound sensor are in good agreement. Especially the grain size of the carburized samples is predicted with good quality. In terms of the as-delivered condition, the grain size appears to be slightly overestimated with around 4 μm. Other investigations such as from Heibel et al. [46] suggest a grain size below 2 μm, which would be in a better agreement with the micrograph. However, since this grain size is on the absolute lower detection limit on the sensor, the results are still satisfactory.

**Figure 11:**
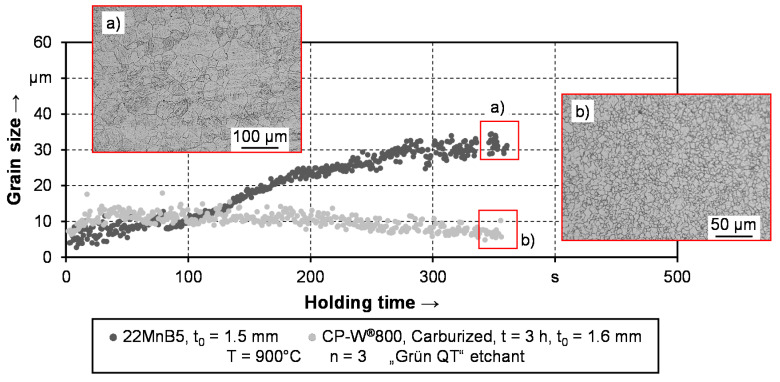
Grain growth of carburized complex phase steel and 22MnB5. The figure includes micrographs of the etched grain size of (**a**) 22MnB5 and (**b**) the carburized complex phase steel.

While the grain size stability of the CP-W^®^800 is well known due to the alloy composition, the results indicate a sufficient grain size stability in the case of prior carburized material as well. To put the result in context, additional experiments are carried out with the conventional hot stamping steel 22MnB5. To demonstrate the advantages of the carburized complex phase steel, the holding time is increased to 6 min. Considering the additional time for the heating process, the overall heat treatment process is slightly longer than conventional austenitization procedures at 900 °C with around 6 min [57] in total. However, minor incidents in the production cycle could result in a prolongation of the furnace lead time. Therefore, the investigation of holding times above conventional dwell times is expedient to evaluate the robustness of the respective alloys in terms of grain size. Since previous findings from Figure 10 suggest that the carburization time has no influence on the grain growth behavior during reaustenitization, only samples being carburized for three h are taken for the comparison. The respective results as well as two exemplarily micrographs are shown in Figure 11. To improve the visibility of the measurement data, no standard deviation is shown in the depiction. In the case of 22MnB5, the average standard deviation is 6.8 μm and in the case of the carburized CP-W^®^800 2.5 μm.

It can be seen, that the 22MnB5 exhibits a significantly more pronounced grain growth behavior compared to the carburized complex phase steel. Upon reaching the austenitization temperature of 900 °C the grain size is in the same range of around five to ten microns. While the grain size stays around 10 μm in the case of the carburized sample, it significantly increases for the 22MnB5 up to around 30 μm. These results are in good agreement with the micrographs depicted. Regarding the grain size of 22MnB5, the investigations of Cai et al. [58] additionally confirm the measurement data in Figure 11. In their study, the authors identify a grain size of 23 μm after an austenitization time of 5 min at 900 °C. This is slightly lower compared to the depicted values in Figure 11 but lies within the range of the average standard deviation of ±6.8 μm. In principle, the 22MnB5 exhibits more scattering regarding the standard deviation, which can be traced back to single large grains that influence the measurement [53], as visible from the micrograph. Based on these results, it can be stated, that 22MnB5 is more prone to grain growth, even at a comparable low austenitization temperature of 900 °C. The grain size does not only influence the mechanical properties but also the phase transformation during quenching [59]. Therefore, improving fine grain stability is one of the current research trends in hot stamping [60].

## 4. Conclusions

In this study, the influence of additional carburization of semi-finished sheets of a complex phase steel before hot stamping on the microstructural evolution during austenitization and quenching was investigated. The results lead to the following conclusions:

- Additional carburization enhances the hardenability of the CP-W^®^800 steel sheets. For a given quenching rate, the phase transformation is shifted to lower temperatures, which is accompanied by the formation of harder phases.
- Due to a gradient in carbon content, the hardenability varies along the sheet thickness.
- Although a multiphase microstructure was present for all quenching rates and material conditions, the hardness of fully martensitic 22MnB5 could partially be exceeded, which is a consequence of the influence of carbon content on martensitic hardness.
- Special care has to be taken in terms of the process design to ensure a sufficient heat transfer during cooling since the mechanical properties exhibited a distinctive dependence from the quenching rates within the investigated range of parameters.
- The in-situ study of the grain growth behavior showed that the additional carburization process leads to an increase in prior austenite grain size from 4 μm to 10 μm after hot stamping. However, grain size stability was not affected by the carburization process. Compared to the conventional 22MnB5 the carburized complex phase steel exhibits higher process robustness in terms of austenite grain size control while having comparable mechanical properties.
- The material behavior of CP-W^®^800 during hot stamping underlines the suitability of the recommended scope of application in small batch size productions and prototyping. The robustness regarding the grain size during austenitization is advantageous in these manufacturing processes where fluctuations in the heat treatment can occur due to a lower degree of automation. Furthermore, cycling time is not an issue and so a sufficient cooling performance of tools can be ensured.

## 5. Outlook

Within this study, the influence of additional carburization treatment on the evolution of microstructure during hot stamping was investigated. Within the investigated parameters, all material conditions exhibited a mixed microstructure after quenching. However, a quantification of the respective phase fraction was not undertaken. Therefore, further research should not only focus on higher cooling rates up to 100 K/s but also include quantitative phase fraction analysis. In the context of faster cooling, occurring latent heat must be compensated in future experiments to guarantee constant quenching rates. Furthermore, the additional analysis should include higher austenitization temperatures, since a heat treatment temperature of 900 °C is in the lower range of relevant carburization and austenitization temperatures.

## Figures and Tables

**Table 1:** Chemical composition of CP-W^®^800 [18] and 22MnB5 [19] in wt.%.

Material	C	Si	Mn	P	S	Al	Ti + Nb	Cr + Mo	V	B
CP-W^®^800	0.14	1.00	2.20	0.080	0.015	0.015–2.0	0.25	1.00	0.20	0.005
22MnB5	0.25	0.40	1.4	0.025	0.010	0.015	--	0.50	--	0.005

**Table 2:** Average quenching rates between 800 °C and 250 °C.

Nominal Quenching Rate ${\overset{\cdot }{T}}_{\mathrm{nom}}$ in K/s	10	30	50	70	100
Non-carburized + simulated hot stamping	10.0	30.0 ± 0.1	49.7 ± 0.3	62.8 ± 1.5	69.8 ± 1.5
Carburized, t = 3 h + simulated hot stamping	10.0	29.9	47.8 ± 0.5	60.6 ± 0.5	67.9 ± 0.9
Carburized, t = 6 h + simulated hot stamping	10.0	29.9	47.8 ± 0.2	61.2 ± 0.3	69.3 ± 0.9

## Data Availability

The datasets generated and analyzed during the current study are available from the corresponding author on reasonable request.
